# Silver‐selenium nanoparticles and selected chemical compounds significantly inhibit grapevine trunk disease pathogens

**DOI:** 10.1002/ps.70110

**Published:** 2025-08-06

**Authors:** Kateřina Štůsková, Tomáš Kiss, Zuzana Bytešníková, Lukáš Richtera, David Gramaje, Ales Eichmeier

**Affiliations:** ^1^ Mendeleum – Institute of Genetics, Mendel University in Brno Lednice Czech Republic; ^2^ Department of Chemistry and Biochemistry Mendel University in Brno Brno Czech Republic; ^3^ Instituto de Ciencias de la Vid y del Vino (ICVV), Consejo Superior de Investigaciones Científicas – Gobierno de La Rioja – Universidad de La Rioja Logroño Spain

**Keywords:** plant protection, *in‐planta* experiment, inhibitory activity, grapevines, statistical evaluation, gene expression

## Abstract

**BACKROUND:**

Grapevine is among the most economically important cultivated crops worldwide, yet it is increasingly threatened by the grapevine trunk disease (GTD) complex. Due to the lack of effective curative treatments for GTDs and the growing need to reduce chemical pesticide use, alternative strategies, such as the application of nanoparticles, are being investigated. In a 2‐year *in planta* study, the inhibitory effects of four chemical compounds and one nanoparticle formulation were evaluated against three serious pathogens associated with GTD complex: *Diaporthe eres* Nitschke, *Diplodia seriata* De Not., and *Eutypa lata* (Pers.) Tul. & C. Tul. Selection of the chemical compounds and nanoparticles was based on their inhibitory effects observed under *in vitro* conditions, as previously reported.

**RESULTS:**

All chemical treatments demonstrated antifungal activity, with inhibition rates ranging from 33.6% to 93.7%. Silver‐selenium nanoparticles exhibited inhibitory effects specifically against *D. eres* and *E. lata*, with inhibition rates between 55.0% and 86.9%. The absence of phytotoxic effects at the applied concentrations was also confirmed for the nanoparticles used in this study.

**CONCLUSION:**

The experimental results demonstrate that the nanoparticles exhibit strong antifungal activity against pathogenic fungi, without causing any detectable phytotoxic effects on grapevines. These findings highlight their potential as a viable alternative to conventional chemical plant protection methods in viticulture. © 2025 The Author(s). *Pest Management Science* published by John Wiley & Sons Ltd on behalf of Society of Chemical Industry.

## Graphical abstract

A 2‐year *in planta* study confirmed antifungal activity of selected chemicals against GTD pathogens. Silver‐selenium nanoparticles inhibited *D. eres* and *E. lata*, but not *D. seriata*. Despite limited spectrum, they show promise as alternatives for targeted GTD control, with no phytotoxic effects observed.

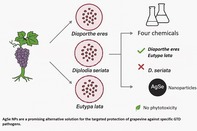

## 1 INTRODUCTION

Grapevine trunk diseases (GTDs) represent a complex of diseases primarily associated with fungal pathogens.^1^, ^2^ The GTD complex encompasses six main diseases: the Esca complex, Eutypa dieback, Botryosphaeria dieback, Phomopsis dieback, Petri disease, and black foot disease.^2^, ^3^ Diagnosing GTDs can be particularly challenging due to the sporadic appearance of symptoms and the overlap of both external and internal manifestations.^4^ The pathogens selected for the in planta experiment in this study were *Diaporthe eres*, *Diplodia seriata*, and *Eutypa lata*, each associated with a distinct form of grapevine trunk diseases. *D. eres* is typically associated with Phomopsis dieback (also known as cane and leaf spot or perennial cankers). This disease is characterized by the formation of black lesions on shoots, followed by progressive wilting and eventual necrosis.^3^, ^5^, ^6^ *D. seriata* is a pathogen responsible for Botryosphaeria dieback, which manifests as necrosis of woody tissues, bark cracking, and gradual dieback of branches.^3^ *E. lata* causes Eutypiosis, characterized by deformation and shortening of shoots, presence of necrotic tissues with distinctive dark streaking in the wood, and progressive death of affected plant parts.^3^, ^4^ GTDs pose significant health and economic threats to grapevines and are present in all major grape‐growing regions worldwide.^7^

The treatment of GTDs remains highly challenging, particularly following the ban of sodium arsenite in 2003 due to its environmental and human health risks.^8^ Complete eradication of GTDs using chemical or biocontrol agents is not possible; therefore, control strategies are primarily aimed at prevention and mitigation of symptoms.^3^, ^9^, ^10^ In the Czech Republic, the protection of grapevines against fungal diseases is strictly regulated and subject to limitations. Although plant protection products targeting GTDs are registered and in use in several regions worldwide, particularly in the Americas and Australia, no such formulation is currently approved for use in the Czech Republic.^11^ Overall, the number of chemical agents approved for GTD control within the European Union remains very limited. Additionally, there is a growing emphasis on the use of biological control agents, particularly the application of fungi from the genus *Trichoderma* ^12^, ^13^, ^14^, ^15^, ^16^, ^17^ or bacteria of the genera *Bacillus* ^18^, ^19^, ^20^ and *Streptomyces*.^19^, ^21^ As a result, chemical solutions are becoming increasingly restricted, and their registration and approval vary across member states.^22^

Nanoparticles (NPs) and nanomaterials (NMs) represent a highly promising and extensively studied field. NMs represent a suitable approach to reducing the use of conventional chemical protection. Within the context of modern agriculture, NPs offer considerable potential to enhance plant resistance to biotic stressors, including pests and pathogens, while simultaneously improving water‐use efficiency, promoting vegetative and reproductive growth, and facilitating plant adaptation to the impacts of climate change. Moreover, their application may significantly reduce the ecological footprint of agricultural systems and contribute to sustainable solutions in the face of the global food security crisis.^23^, ^24^, ^25^, ^26^ Nanomaterials have increasingly attracted attention in the field of agriculture for their potential application in the sustainable remediation of soil degradation. Due to their high surface area‐to‐volume ratio, adjustable physicochemical properties, and enhanced reactivity, they have recently been identified as promising candidates for nano‐enabled fertilizers. These nanofertilizers exhibit superior bioavailability and improved nutrient delivery efficiency, allowing for more effective plant uptake compared to conventional bulk formulations. Their application may thus contribute not only to soil restoration but also to increased nutrient use efficiency and reduced environmental impact.^27^, ^28^, ^29^

In a previously published study, an *in vitro* assay was conducted to evaluate the antifungal efficacy of newly synthesized silver selenium (AgSe) and copper selenium (CuSe) NPs, as well as mono‐elemental NPs of silver (Ag NPs), copper (Cu NPs), and selenium (Se NPs).^1^ In addition to selected chemical agents including sodium arsenite, 8‐hydroxyquinoline, silver nitrate, colloidal silver, Altron Silver fertilizer, and the silver thiosulfate complex (NH_4_)_3_/Ag(S_2_O3)_2_/. These compounds were tested against three prevalent grapevine trunk pathogens (GTPs) – *D. eres*, *E. lata*, and *D. seriata*. Among the tested agents, silver nitrate and AgSe NPs exhibited the most pronounced antifungal activity. These results highlight the potential of Ag‐based nanomaterials, particularly AgSe NPs, as effective antifungal agents against GTPs.^1^

Based on the results of the previous *in vitro* study,^1^ the current *in planta* experiment included a selection of treatments: sodium arsenite, hydroxyquinoline sulfate (8‐HCH), silver nitrate, silver thiosulfate complex, and AgSe NPs. Each compound was applied at the concentrations that exhibited the highest inhibitory effect in the earlier *in vitro* experiment.^1^ We hypothesized that (i) the selected chemical compounds and NPs would exhibit antifungal activity against GTD pathogens, and (ii) their application would alter the gene expression profile of grapevine.

## 2 MATERIALS AND METHODS

### 2.1 Chemical substances and nanomaterial for inhibition experiment

The chemical compounds and NPs used in this study are listed in Table 1. Sodium arsenite, silver nitrate, and silver thiosulfate complex were purchased from Merck KGaA (Darmstadt, Germany), while 8‐HCH was obtained from Carl Roth GmbH & Co. KG (Karlsruhe, Germany). Sodium arsenite was tested at a concentration of 6.50 g l^−1^, and 8‐HCH at 0.05 g l^−1^. Silver nitrate was applied at a concentration of 1.00 g l^−1^. The silver thiosulfate complex was tested at 100% concentration, containing 5.15 g l^−1^ Ag, 105.20 g l^−1^ AgN, and 217.50 g l^−1^ AgS. The AgSe NPs were newly synthesized by the Department of Chemistry and Biochemistry at Mendel University in Brno. Details of the synthesis are described by Štůsková *et al*. (2022).^1^ The AgSe NPs, tested at 100% concentration, contained 2.59 g l^−1^ Ag and 0.90 g l^−1^ Se.

**Table 1:** Chemical compounds and nanoparticles, including their active substances and concentrations, used in the in planta experiment to evaluate their inhibitory effect against the pathogens *D. eres*, *D. seriata*, and *E. lata*

Name	Active ingredient	Concentration	Producer
Sodium arsenite	Sodium arsenite	6.50 g l^−1^	Merck KGaA, Darmstadt, Germany
8‐Hydroxyquinoline	8‐hydroxyquinoline	0.05 g l^−1^	Carl Roth GmbH & Co. KG, Karlsruhe, Germany
Silver nitrate	Ag^+^	1.00 g l^−1^	Merck KGaA, Darmstadt, Germany
Silver thiosulfate complex	Ag^+^	5.15 g l^−1^Ag, 105.20 g l^−1^AgN, 217.50 g l^−1^AgS	Merck KGaA, Darmstadt, Germany
AgSe NPs	Ag, Se	2.59 g l^−1^ Ag, 0.90 g l^−1^ Se	Department of Chemistry and Biochemistry, Mendel University, Brno, Czech Republic

### 2.2 Fungal isolates

The selected fungal pathogens are listed in Table 2. All strains are maintained in culture collections at Mendeleum – Institute of Genetics, Mendel University in Brno (Lednice, Czech Republic) or CBS‐KNAW (Utrecht, Netherlands). Three GTD pathogens were selected: *D. eres* CPC 28220,^6^ *D. seriata* OCR120,^30^ and *E. lata* CS_12_27_4_KO.^31^ *D. eres* and *E. lata* were isolated from grapevine wood, while *D. seriata* was isolated from walnut wood. All three pathogens are polyphagous and known to infect a wide range of plant hosts, including grapevines, pears, walnuts, blackberries, and mulberries.^30^, ^32^, ^33^

**Table 2:** Selected pathogens causing diseases within the grapevine trunk disease (GTD) complex and their origin, on which the inhibitory effects of selected chemical compounds and nanoparticles were tested in the in planta experiment

Pathogen	Name of isolate	Host plant	Accession number	Source
*Diaporthe eres*	CPC28220	Grapevine	MG281023	Guarnaccia *et al*., 2018
*Diplodia seriata*	OCR120	Walnut	MK431136	Eichmeier *et al*., 2020
*Eutypa lata*	CS_12_27_4_KO	Grapevine	KY270972	Baránek *et al*., 2018

### 2.3 Inhibitory activity

The *in planta* experiment was conducted over two consecutive years (2021 and 2022) in a net house at Mendeleum – Institute of Genetics, Mendel University in Brno, Lednice, Czech Republic. The grapevine cultivar Sauvignon Blanc, grafted onto SO4 rootstock, was selected for the experiment due to its known high susceptibility to GTD pathogens.^34^, ^35^, ^36^ To simulate infection and evaluate the inhibitory effect of the treatments, the stem of each grafted vine was carefully peeled in the upper part, approximately 5–10 cm below the apex, using a sterile scalpel to expose the underlying tissue. At this site, a 3 mm diameter hole was created with a cork borer for the application of mycelial plugs.^37^ Chemicals or NPs were applied directly into the wound using a sterile brush. Sterile water served as the positive control. Each treatment was applied to four plants (replicates). Fungal discs (3 mm in diameter) were excised from active pathogen cultures and aseptically placed onto the treated wound sites. The inoculation site was covered with moistened cotton wool and sealed with Parafilm to maintain humidity. The grapevines were planted in containers and maintained under net house conditions, with regular monitoring and irrigation as needed. After 6 months, grapevines were removed from the containers and roots were thoroughly washed with tap water. Measurement included total grapevine height (stem height + length of the longest cane) and root length. To assess internal disease progression, bark at the inoculation site was removed and wood necrosis was measured. Necrosis was expressed as a percentage of stem height using the formula:

$$x=\frac{n\times 100}{s},$$

where *x* = percentage of necrosis, *n* = necrosis size (cm), and *s* = stem height (cm). Pathogens were re‐isolated from the edge of necrotic tissues to confirm infection. Additionally, RNA was subsequently isolated from the wood for subsequent gene expression analysis. The percentage of inhibition was calculated using the formula:

$$I=\left(\left(r_{c}-r_{t}\right)/r_{c}\right)\times 100,$$

where *I* is the percentage of inhibition, *r*_c_ is the average percentage of necrosis in the control group, and *r*_t_ is the average percentage of necrosis in the treated group.^1^

### 2.4 Statistical analysis

Statistical analysis was performed using Statistica 14 CZ (StatSoft, Prague, Czech Republic). For each treatment, four plants were planted resulting in four numerical measurements per parameter: plant height (cm), root length (cm), and necrosis size (cm). A one‐way analysis of variance (ANOVA) was conducted separately for each parameter at a significance level of *P* = 0.05. When significant differences were detected, a *post hoc* least significant difference (LSD) test was applied at the same significance threshold (*P* = 0.05). The LSD test was used to assess the significance of differences between each treatment and the control.

### 2.5 Pathogen re‐isolation and identification

For pathogen re‐isolation, necrotic wood was surface‐sterilized by soaking in 1% sodium hypochlorite for 3 min, rinsed three times with distilled water, and then cut into small pieces using a sterile scalpel. Wood fragments were aseptically transferred onto potato dextrose agar (PDA; HiMedia, Čaderský‐Envitek, Brno, Czech Republic) supplemented with streptomycin (HiMedia, Čaderský‐Envitek, Brno, Czech Republic) at a concentration of 100 mg/L to inhibit bacterial growth. Emerging fungal colonies were subcultured onto fresh PDA plates to obtain pure cultures. These pure cultures were then grouped into three categories based on morphological characteristics: the first group included cultures morphologically consistent with *Diaporthe*, the second with *Diplodia*, and the third with *Eutypa*. One representative culture from each group was selected, and mycelium was collected for DNA extraction and subsequent sequencing to confirm the genus. To identify the reisolated fungi at the genetic level, genomic DNA was extracted from selected pure cultures using the NucleoSpin Tissue Kit (Macherey‐Nagel, Düren, Germany). Amplification of the internal transcribed spacer (ITS) region was performed using the universal fungal primers ITS1 (5’‐TCC GTA GGT GAA CCT GCG G‐3′) and ITS4 (5’‐TCC TCC GCT TAT TGA TAT GC‐3′).^38^ *Diaporthe* was identified to the species level based on beta‐tubulin (tub2) and translation elongation factor 1‐alpha (tef1) sequences, while *Diplodia* and *Eutypa* were identified based on the tub2 sequence, following Eichmeier *et al*. (2020).^30^ Sample preparation for sequencing was carried out using the Mix2Seq Kit (Eurofins Genomics, Ebersberg, Germany), and sequencing was performed by Eurofins Genomics. Resulting sequences were compared to reference sequences using the BLAST algorithm^39^ using GenBank NCBI to determine fungal identity.

### 2.6 Gene expression analysis

Gene expression analysis was performed only in the second year of the experiment, following the significant inhibition effects observed during the first year. To gain deeper insights into the interaction between the pathogen, the plant, and the applied substance, the second‐year analysis focused on the plant's molecular response.

Four marker genes were selected for gene expression analysis: actin (*VvACT*), non‐expresser of pathogenesis‐related gene 1 (*VvNPR1*), stilbene synthase (*VvSTS*), and acidic class IV chitinase (*VvChit4c*). The *VvACT* gene served as a housekeeping gene to normalize the expression levels of the target genes. Gene expression was evaluated using real‐time PCR (qPCR). Primer sequences are listed in Table 3. Total RNA was extracted using the Spectrum™ Plant Total RNA Kit (Sigma‐Aldrich, St. Louis, Missouri, USA). RNA concentrations were measured using a SpectrostarNano (BMG Labtech, Ortenberg, Germany) and adjusted to a uniform concentration of 90 ng/μL using sterile nuclease‐free water (Ambion, Pleasanton, California, USA). Reverse transcription was performed following the protocol described by Eichmeier *et al*. (2010),^40^ using the RevertAid RT Reverse Transcription Kit (Thermo Fisher Scientific, Waltham, Massachusetts, USA), resulting in complementary DNA (cDNA) samples. qPCR was carried out using Genaxxon mastermix (Genaxxon Bioscience GmbH, Ulm, Germany) on a qTower3 real‐time PCR cycler (Analytik Jena, Jena, Germany), with gene‐specific thermal cycling protocols outlines in Table S1. Fluorescence signals and data analysis were processed using qPCRsoft 3.4 software (Analytik Jena). Statistical analysis of normalized gene expression levels was conducted using Statistica 14 CZ. A gene was considered upregulated when its normalized expression exceeded a 2‐fold increase, and downregulated when it dropped below 0.5‐fold, relative to the untreated controls.

**Table 3:** Primer sequences for selected genes involved in the grapevine pathogen–host interaction. This analysis was used to evaluate gene expression in plants inoculated with grapevine trunk disease (GTD) pathogens and subjected to various treatments with chemical compounds and nanoparticles

Gene	Accession number	Forward primer 5′ → 3'	Reverse primer 3′ → 5'
VvACT	AF369524^a^	TGCTATCCTTCGTCTTGACCTTG	GGACTTCTGGACAACGGAATCTC
VvNPR1	GSVIVT00016536001^b^	GGAATTCGATGTTGGGTACG	GCAACCTTGTCAAGAATGTCC
VvSTS	DQ366301^a^	CATCAAGGGTGCTATGCAGGT	TCAGAGCACACCACAAGAACTCG
VvChit4c	AY137377^a^	TCGAATGCGATGGTGGAAA	TCCCCTGTCGAAACACCAAG

^a^ GenBank accession number.

^b^ Genoscope Grape Genome Browser number.

## 3 RESULTS

### 3.1 Inhibitory activity

The measured values of grapevine height (cm), root length (cm), and necrosis size (%) are shown in Table 4, while inhibition percentages are presented in Table S2.

**Table 4:** Measured values of grapevines height and root length, as well as the calculated percentage of necrosis in grapevine grapevines inoculated with grapevine trunk disease (GTD) pathogens and treated with selected chemical compounds and nanoparticles as part of the in planta experiment

			Sodium arsenite	8‐Hydroxy‐quinoline	Silver nitrate	Silver thiosulfate complex	AgSe NPs	Control (sterile water)
*Diaporthe eres*	Grapevine height (cm)	2021	65.67	82.25	83.83	84.33^a^	74.25	61.67
		2022	110.25	125.88	102.25	138.00	110.75	105.75
	Root length (cm)	2021	32.67^a^	34.25^a^	40.83^a^	33.17^a^	32.63^a^	19.67
		2022	35.75^a^	34.00	29.75	31.50	29.75	26.50
	Necrosis (%)	2021	22.40^a^	30.71^a^	7.71^a^	8.81^a^	7.65^a^	58.21
		2022	52.34^a^	26.88^a^	41.69^a^	19.80^a^	31.97^a^	98.39
*Diplodia seriata*	Grapevine height (cm)	2021	55.25	63.00	64.83	64.75	91.17^a^	60.63
		2022	106.00	110.00	105.75	105.00	118.50	110.50
	Root length (cm)	2021	30.00	29.00	37.17	31.00	37.67	26.88
		2022	34.50^a^	29.75	25.00	39.25^a^	33.25^a^	24.75
	Necrosis (%)	2021	27.46^a^	3.51^a^	10.98^a^	8.67^a^	27.90	55.26
		2022	43.39^a^	28.52^a^	54.61^a^	46.53^a^	65.03	84.69
*Eutypa lata*	Grapevine height (cm)	2021	91.00	63.67	72.00	56.67	N/A	73.00
		2022	126.25^a^	96.00	94.50	106.50	115.00^a^	83.50
	Root length (cm)	2021	38.00	25.33	35.00	28.00	N/A	32.83
		2022	30.50^a^	25.75	36.25^a^	27.25	32.00^a^	23.50
	Necrosis (%)	2021	19.76^a^	4.74^a^	8.97^a^	10.72^a^	N/A	34.11
		2022	55.80^a^	12.45^a^	48.41^a^	29.33^a^	37.83^a^	84.08

*Note*: NPs nanoparticles. N/A not analyzed (due to the death of 3 out of 4 seedlings, statistical analysis could not be performed).

^a^ Value showing a significant difference from control.

In grapevine inoculated with *D. eres*, a statistically significant increase in grapevine height was observed only in 2021 for the treatment with silver thiosulfate complex, which reached 84.33 cm compared to 61.67 cm in the sterile water control. However, in 2022, no treatment, including silver thiosulfate complex, significantly affected grapevine height. Conversely, root length was significantly influenced by all treatments in the first year, with values ranging from 32.63 to 40.83 cm, compared to 19.67 cm in the control. In the second year, only sodium arsenite significantly increased root length, with a value of 35.75 cm *versus* 26.50 cm in the control. All tested chemical substances and nanoparticles had a significant effect on necrosis size and inhibition percentage in both years. In 2021, necrosis sizes for sodium arsenite, 8_HCH, silver nitrate, silver thiosulfate complex, and AgSe NPs were 22.40%, 30.71%, 7.71%, 8.81%, and 7.65%, respectively, compared to 58.21% in the control. The corresponding inhibition percentages were 61.51% for sodium arsenite, 47.24% for 8‐HCH, 86.75% for silver nitrate, 84.87% for silver thiosulfate complex, and 86.85% for AgSe NPs. In 2022, necrosis sizes were 52.34%, 26.88%, 41.69%, 19.80%, and 31.97% for sodium arsenite, 8‐HCH, silver nitrate, silver thiosulfate complex, and AgSe NPs, respectively, while the control showed 98.39% necrosis. Corresponding inhibition percentages were 46.80% for sodium arsenite, 72.68% for 8‐HCH, 57.63% for silver nitrate, 79.87% for silver thiosulfate complex, and 67.51% for AgSe NPs (Fig. S1).

In grapevines inoculated with *D. seriata*, a statistically significant increase in grapevine height was observed only in 2021 for the treatment with AgSe NPs, where the average height reached 91.17 cm compared to 60.63 cm in the control group treated with sterile water. In 2022, none of the treatment groups had a significant effect on grapevine height. Root length was not significantly affected by any of the selected chemicals or NPs in the first year. However, in 2022, sodium arsenite (34.50 cm), silver thiosulfate complex (39.25 cm), and AgSe NPs (33.25 cm) significantly increased root length relative to the control (24.75 cm). Necrosis size and inhibition percentage were significantly affected by all tested chemical treatments in both years. However, neither of the NP treatments has a statistically significant effect on these parameters in either year. In 2021, necrosis sizes for sodium arsenite, 8‐HCH, silver nitrate, and silver thiosulfate complex were 27.46%, 3.51%, 10.98%, and 8.67%, respectively, compared to 55.26% in the control. Corresponding inhibition percentages were 50.30% for sodium arsenite, 93.65% for 8‐HCH, 80.13% for silver nitrate, and 84.31% for silver thiosulfate complex. In 2022, necrosis sizes for sodium arsenite, 8‐HCH, silver nitrate, and silver thiosulfate complex were 43.39%, 28.52%, 54.61%, and 46.53%, respectively, compared to 84.69% in the control. The corresponding inhibition percentages were 48.77% for sodium arsenite, 66.32% for 8‐HCH, 35.52% for silver nitrate, and 45.06% for silver thiosulfate complex (Fig. S2).

In grapevines inoculated with *E. lata*, the AgSe NPs treatment was not evaluated in the first year due to dieback of three out of four grapevines, which precluded statistical analysis. In 2021, none of the chemical treatments significantly affected grapevine height. However, in 2022, significant increases in grapevine height were observed with sodium arsenite (126.25 cm) and AgSe NPs (115.0 cm), compared to the control (83.50 cm). Root length was not significantly affected by any treatment in 2021. In contrast, in 2022, root length was significantly increased by sodium arsenite (30.50 cm), silver nitrate (36.25 cm), and AgSe NPs (32.00 cm), compared to the control (23.50 cm). Necrosis size and corresponding inhibition percentage were significantly affected by all tested chemicals in the first year. Necrosis sizes were 19.76% for sodium arsenite, 4.74% for 8‐HCH, 8.97% for silver nitrate, and 10.72% for silver thiosulfate complex, compared to 34.11% in the control. Corresponding inhibition percentages were 42.08% for sodium arsenite, 86.11% for 8‐HCH, 73.72% for silver nitrate, and 63.58% for silver thiosulfate complex. In 2022, necrosis sizes were 55.80% for sodium arsenite, 12.45% for 8‐HCH, 48.41% for silver nitrate, 29.33% for silver thiosulfate complex, and 37.83% for AgSe NPs, while the control recorded 84.08%. The inhibition percentages were 33.64% for sodium arsenite, 85.18% for 8‐HCH, 42.43% for silver nitrate, 65.11% for silver thiosulfate complex, and 55.01% for AgSe NPs (Fig. S3).

### 3.2 Re‐isolation of pathogens

The number of plants from which pathogens were successfully re‐isolated is presented in Table 5. The re‐isolation method confirmed the presence of all inoculated pathogens, verifying successful infection of the grapevines. In both years, the target pathogens were re‐isolated from 3 to 4 plants per treatment (75–100%), thereby fulfilling Koch's postulates.

**Table 5:** Re‐isolation of pathogens

		Number of plants					
		*Diaporthe*		*Diplodia*		*Eutypa*	
Substance	Applied pathogen	2021	2022	2021	2022	2021	2022
Sodium arsenite	*Diaporthe eres*	3	4	0	0	0	0
	*Diplodia seriata*	0	1	3	3	1	0
	*Eutypa lata*	0	1	0	0	4	4
8‐Hydroxyquinoline	*Diaporthe eres*	3	4	1	0	0	0
	*Diplodia seriata*	0	0	3	4	0	0
	*Eutypa lata*	0	1	0	0	3	3
Silver nitrate	*Diaporthe eres*	3	4	1	0	0	1
	*Diplodia seriata*	0	1	4	3	0	0
	*Eutypa lata*	0	0	1	0	3	4
Silver thiosulfate complex	*Diaporthe eres*	4	4	1	0	0	0
	*Diplodia seriata*	1	0	4	4	1	0
	*Eutypa lata*	0	0	1	0	4	4
AgSe NPs	*Diaporthe eres*	4	4	0	1	1	0
	*Diplodia seriata*	0	1	4	4	0	1
	*Eutypa lata*	N/A	0	N/A	0	N/A	4
Control (sterile water)	*Diaporthe eres*	4	3	1	1	0	0
	*Diplodia seriata*	0	1	4	4	1	0
	*Eutypa lata*	1	1	1	0	3	4

*Note*: NPs nanoparticles. N/A not analyzed (due to the death of 3 out of 4 grapevines, statistical analysis could not be performed).

### 3.3 Gene expression analysis

In the analysis of gene expression, three genes involved in the plant‐pathogen response were monitored. The *VvNPR1* gene was downregulated in response to 8‐HCH and silver nitrate.

**Figure 1:**
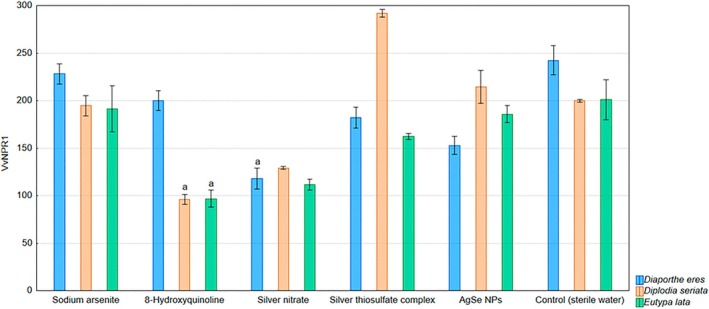
Expression levels of the gene VvNPR1 monitored in the in planta experiment evaluating the inhibitory effects of selected chemical compounds and nanoparticles against the grapevine trunk disease pathogens *D. eres*, *D. seriata*, and *E. lata*: a – the gene was downregulated. There was no upregulation of the VvNPR1 gene in any of the treated groups.

8‐HCH downregulated *VvNPR1* in grapevines inoculated with the pathogens *D. seriata* and *E. lata*, while in the case of silver nitrate, downregulation was observed in *D. eres*. In other treatments, the expression of this gene was not affected (Fig. 1).

**Figure 2:**
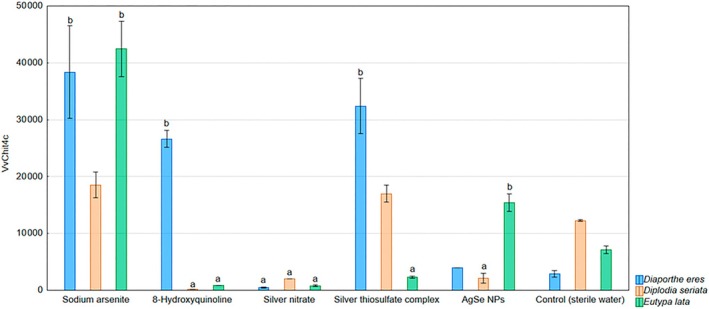
Expression levels of the gene VvChit4c monitored in the in planta experiment evaluating the inhibitory effects of selected chemical compounds and nanoparticles against the grapevine trunk disease pathogens *D. eres*, *D. seriata*, and *E. lata*: a – the gene was downregulated; b – the gene was upregulated.

The *VvChit4c* gene was upregulated in grapevines treated with sodium arsenite and inoculated with *D. eres* and *E. lata*. In grapevines treated with 8‐HCH and inoculated with *D. seriata* and *E. lata*, downregulation was observed, whereas in the case of *D. eres*, *VvChit4c* was upregulated. The silver nitrate treatment exhibited downregulations for all tested pathogens. Silver thiosulfate complex increased expression in grapevines inoculated with *D. eres* but decreased expression in those inoculated with *E. lata*. AgSe NPs reduced *VvChit4c* expression in grapevines inoculated with *D. seriata*, whereas in those inoculated with *E. lata*, expression was increased (Fig. 2).

**Figure 3:**
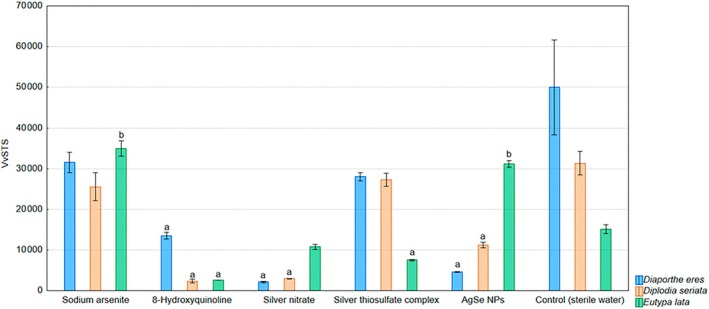
Expression levels of the gene VvSTS monitored in the in planta experiment evaluating the inhibitory effects of selected chemical compounds and nanoparticles against the grapevine trunk disease pathogens *D. eres*, *D. seriata*, and *E. lata*: a – the gene was downregulated; b – the gene was upregulated.

The last evaluated gene expression was the stilbene synthesis gene *VvSTS*. The expression of this gene was significantly reduced by 8‐HCH, silver nitrate, silver thiosulfate complex, and AgSe NPs. 8‐HCH downregulated expression in all three tested pathogens, while silver nitrate did not affect expression in grapevines inoculated with *E. lata*. Silver thiosulfate complex downregulated VvSTS expression in grapevines inoculated with *E. lata*. AgSe NPs reduced expression in grapevines inoculated with *D. eres* and *D. seriata*, whereas in grapevines inoculated with *E. lata*, expression was significantly increased. In the case of *E. lata*, expression was also increased by sodium arsenite application (Fig. 3).

This study also confirmed that the nanoparticles used did not exhibit any phytotoxicity at the applied concentrations.

## 4 DISCUSSION

The application of nanotechnology in agriculture has presented a major revolution. The increasing pressure to intensify crop yield is focusing on the application of nano‐biotechnologies in agriculture to improve the food market in a sustainable manner. This study evaluated the *in planta* antifungal effects of four chemical compounds and one nanoparticle formulation over 2 years against key GTD pathogens: *Diaporthe eres*, *Diplodia seriata*, and *Eutypa lata*. All tested treatments showed inhibitory effects, with silver‐selenium nanoparticles proving particularly effective against *D. eres* and *E. lata*, and without phytotoxic effects. The findings support the potential of nanoparticles as an environmentally friendly alternative to conventional plant protection in viticulture.

In our study, sodium arsenite significantly inhibited all tested GTD pathogens. This inhibitory effect has also been reported in other studies. Trouvelot *et al*. (2023)^41^ observed that grapevines treated with sodium arsenite did not develop foliar symptoms of Esca. Moreover, their study confirmed the impact of sodium arsenite on the metabolome, including the production of defensive secondary metabolites and histological changes in the interaction zone where the pathogen challenge occurred. In addition to its direct antifungal activity, sodium arsenite has been shown to stimulate the production of plant secondary metabolites associated with plant defense, particularly within woody tissues. This stimulatory effect on host tolerance and metabolic responses was also reported by Songy *et al*. (2019).^42^ Moreover, Bruez *et al*. (2021)^43^ demonstrated the inhibitory effect of sodium arsenite on *Fomitiporia mediterranea*, basidiomycete species involved in the Esca complex. In their study on the grapevine wood mycobiome, *F. mediterranea* showed a relative abundance of 63–94% in the control group, compared to only 1–40% in the group treated with sodium arsenite. A reduction in *D. seriata* and *E. lata* was also observed in the treated plants; however, it was not as pronounced as in the case of *F. mediterranea*. For *D. eres*, our study was the first to confirm the inhibitory effect of sodium arsenite against this pathogen.

The antifungal activity of 8‐HCH against GTD pathogens has also been confirmed in previous studies. Castillo‐Pando *et al*. (1997)^44^ reported that 8‐HCH, applied at a concentration of 1.5 g l^−1^ to one‐year‐old grapevine canes infected with *Diaporthe ampelina*, which causes Phomopsis dieback, reduced pycnidia viability to 0.1%, compared to 100% viability in the untreated control. Similarly, Fourie and Halleen (2006)^45^ observed a reduction in GTD pathogen incidence to 13.1% in grapevines treated with Chinosol (8‐HCH), in contrast to 26.4% in the untreated controls. In addition, Souza *et al*. (2021)^46^ demonstrated the efficacy of a synthetic derivative of 8‐HCH PH 151‐8‐hydroxyquinoline‐5‐(N‐4‐chlorophenyl) sulfonamide against *Ilyonectria liriodendri* in an *in vivo* experiment. At a concentration of 0.625 g l^−1^, PH 151 resulted in only 17% re‐isolation of the pathogen, whereas the commercial fungicide Mancozeb, used at the same concentration, showed a re‐isolation rate of 82%. The positive control showed 90% re‐isolation, highlighting the superior inhibitory effect of the 8‐HCH derivative.

Silver nitrate also demonstrated inhibitory activity against GTD pathogens in our study; however, its use on grapevine has not been previously reported. Nonetheless, several studies have confirmed its antimicrobial properties against other plant pathogens. For example, Ganiyu *et al*. (2024)^47^ reported that foliar application of silver nitrate at a concentration of 0.1 g l^−1^ on tomato plants significantly reduced the incidence of tomato wilt caused by *Fusarium oxysporum* f.*sp. lycopersici* to 10% compared to 89.90% in untreated controls. Similary, Oyelakin *et al*. (2023)^48^ observed that application of silver nitrate 16.99 g l^−1^ on tomato plants (cv. Roma VF) reduced the incidence of late blight caused by *Phytophthora infestans* from 53.33% in control plants to 33.30% in treated plants. In the 82B tomato cultivar, the reduction was even more substantial, from 60% in controls to 23.33% in treated plants. Additionally, Jo *et al*. (2009)^49^ demonstrated significant antifungal effects of silver nitrate against *Bipolaris sorokiniana* Shoemaker and *Magnaporthe grisea* in perennial ryegrass (*Lolium perenne* L.). When applied at a concentration of 0.05 g l^−1^, silver nitrate reduced leave damage to 7% compared to 70% in the sterile water control.

In our study, the *in planta* experiment demonstrated that the silver thiosulfate complex exhibited statistically significant inhibitory activity against all three tested GTD pathogens: *D. eres*, *D. seriata*, and *E. lata*. To our knowledge, no previous studies have investigated the antifungal activity of the silver thiosulfate complex against plant‐pathogenic fungi, making this one of the first reports highlighting its potential in plant disease management.

AgSe NPs demonstrated significant inhibitory activity against *D. eres* and *E. lata*, but not against *D. seriata*. Currently, no studies have directly assessed the efficacy of AgSe NPs against grapevine pathogens; however, several studies confirm the antifungal effects of individual Ag and Se NPs on fungal pathogens affecting other cultivated crops. Malandrakis *et al*. (2019)^50^ reported that Ag NPs at a concentration of 0.001 g l^−1^, achieved 100% inhibition of *Botrytis cinerea* on plum (*Prunus domestica* L.) fruits. Similarly, Jebril *et al*. (2020)^51^ evaluated the *in vivo* efficacy of Ag NPs at 0.02 g l^−1^, applied *via* soil irrigation after inoculation with *Verticillium dahlia* in eggplant (*Solanum melongena* L.) and observed an 87% reduction in diseases severity compared to untreated controls. The antifungal potential of Se NPs has also been reported. Hashem *et al*. (2021)^52^ found that Se NPs applied at 0.005 g l^−1^ reduced the disease index in faba bean (*Vicia faba* L.) plants infected with *Rhizoctonia solani* from 88% (control) to 20%. Likewise, Nandini *et al*. (2017)^53^ demonstrated that treating pearl millet (*Cenchrus americanus*) seeds with Se NPs (0.1 g l^−1^) significantly reduced the incidence of downy mildew caused by *Sclerospora graminicola*, from 93% in untreated plants to 49% in treated ones.

The expression patterns of three defense‐related genes (VvNPR1, VvChit4c, and VvSTS) revealed distinct responses depending on the applied treatment and the GTD pathogen involved, indicating a complex interaction between the grapevine defense system and the applied compounds.

The gene VvNPR1, a key regulator of systemic acquired resistance, was downregulated by 8‐HCH and silver nitrate, suggesting a possible suppression of salicylic acid‐mediated signaling. Notably, 8‐HCH suppressed VvNPR1 expression in plants inoculated with *D. seriata* and *E. lata*, while silver nitrate had a similar effect in plants inoculated with *D. eres*. In other treatments, VvNPR1 expression remained stable, indicating that these compounds selectively affect signaling pathways depending on the pathogen. The effect of chemical compounds on the upregulation of VvNPR1 expression has also been observed in other studies. An increase in VvNPR1 expression was recorded following the application of benzothiadiazole, both in grapevine cell cultures^54^ and in *Vitis vinifera* cv. Chardonnay plants.^55^ A transient increase in VvNPR1 expression was also observed following postharvest treatment of grape berries with β‐aminobutyric acid.^56^

The expression of VvChit4c, which encodes a class IV chitinase and is associated with fungal cell wall degradation, showed greater variability. Sodium arsenite significantly upregulated VvChit4c expression in grapevines inoculated with *D. eres* and *E. lata*, suggesting activation of defense mechanisms. In contrast, 8‐HCH led to downregulation in *D. seriata* and *E. lata*‐inoculated plants, but induced expression in *D. eres*, highlighting a possible pathogen‐specific modulation. Silver nitrate consistently suppressed VvChit4c expression across all pathogens, potentially indicating an overall suppressive effect on this defense pathway. Interestingly, silver thiosulfate complex and AgSe NPs produced mixed responses depending on the pathogen, further supporting the hypothesis that the grapevine response is highly specific and not solely dependent on the treatment itself. Differential modulation of chitinase gene expression following chitosan treatment and pathogen infection was also confirmed by the study of Martín *et al*. (2023).^57^ Application of chitosan to asymptomatic grapevine plants did not result in any significant changes in the expression of chitinase‐encoding genes. In contrast, in plants exhibiting symptoms of Esca disease complex, chitosan treatment led to a pronounced downregulation of these genes. This finding, consistent with the results of our study, suggests that the effect of chitosan is pathogen‐dependent and that, in symptomatic plants, it may modulate defense pathways toward suppression of genes associated with pathogen cell wall degradation.

Finally, the VvSTS gene, responsible for stilbene synthesis, was generally downregulated by most treatments, including 8‐HCH, silver nitrate, silver thiosulfate complex, and AgSe NPs. Stilbenes are known phytoalexins involved in pathogen resistance, and their reduced expression may suggest a possible trade‐off or redirection of defense signaling. However, in *E. lata*‐inoculated plants, AgSe NPs and sodium arsenite induced VvSTS expression, suggesting a potential enhancement of stilbene‐based defense under certain conditions. According to the study by Songy *et al*. (2019),^42^ one of the factors influencing the modulation of VvSTS gene expression may be the phenological stage of the grapevine. Following the application of sodium arsenite to grafted *Vitis vinifera* cv. Tempranillo plants, a more than twofold upregulation of the VvSTS gene was observed during the flowering stage. In contrast, no significant changes in gene expression were detected during the berry maturity stage. These results suggest that the effect of chemical treatment may be strongly dependent on the developmental stage of the plant.

Together, these results indicate that the tested compounds can differentially modulate grapevine defense‐related gene expression in a pathogen‐dependent manner. While some treatments stimulated specific defense pathways, others appeared to suppress them, underlining the importance of context in evaluating the potential of alternative disease management strategies. Further research should focus on linking these molecular responses to actual disease progression and field efficacy to better understand their protective potential.

## 5 CONCLUSION

The *in planta* experiment confirmed the inhibitory activity of selected chemical compounds against GTD pathogens, specifically *D. eres*, *D. seriata*, and *E. lata*. The newly synthesized AgSe NPs exhibited significant inhibitory effects against *D. eres* and *E. lata*. However, no significant inhibition was observed against *D. seriata*. Although AgSe NPs did not demonstrate efficacy against all the tested pathogens, the results suggest that these nanoparticles hold promise as a potential alternative for the plant protection against specific fungal pathogens. Further research is needed to optimize their formulation and broaden their spectrum of activity. The nanoparticles tested in this study showed no signs of phytotoxicity at the concentrations applied, nevertheless, further research using the method of treating inoculated pruning wounds will be necessary.

## AUTHOR CONTRIBUTIONS

A. Eichmeier, D. Gramaje and K. Štůsková designed the scheme of the experiment. K. Štůsková established and evaluated the *in‐planta* experiment, performed the statistical analysis and wrote the main part of the text. T. Kiss and K. Štůsková designed the gene expression analysis procedure, performed real‐time PCR and evaluated the effect of treatments on the expression profile of grapevines. Z. Bytešníková and L. Richtera prepared the NPs used. A. Eichmeier and D. Gramaje approved the scheme of the experiment, supervised the course of the experiment, and edited the text.

## Supporting information


**Data S1.** Supporting Information.

## Data Availability

The data that support the findings of this study are available from the corresponding author upon reasonable request.
